# Case report: Pulmonary artery sarcoma diagnosed through rare brain metastases

**DOI:** 10.3389/fonc.2024.1394708

**Published:** 2024-05-15

**Authors:** Na Tan, Zhiqiang Ouyang, Xirui Duan, Xinyan Zhou, Yu Zhu, Jixiang Chu, Dan Luo, Hai-Long Dai, Chengde Liao

**Affiliations:** ^1^ Department of Radiology, Kunming Yan’an Hospital (Yan’an Hospital Affiliated to Kunming Medical University, Kunming, China; ^2^ Department of Radiology, Yunnan Cancer Hospital, The Third Affiliated Hospital of Kunming Medical University, Kunming, China; ^3^ Department of Pathology, Yunnan Cancer Hospital, The Third Affiliated Hospital of Kunming Medical University, Kunming, China; ^4^ Department of Cardiovascular Medicine, Kunming Yan’an Hospital (Yan’an Hospital Affiliated to Kunming Medical University), Kunming, China

**Keywords:** case report, pulmonary artery sarcomas, brain metastases, multidisciplinary team, pulmonary hypertension

## Abstract

We present the case of a 33-year-old male referred across several hospitals because of suspected chronic thromboembolic pulmonary hypertension (CTEPH). Initially admitted in October 2022 for a recurrent, severe cough and diagnosed with CTEPH, he received anticoagulant therapy. However, his symptoms worsened, necessitating a transfer to another facility for thrombolysis treatment. Following an episode of syncope, an MRI scan revealed a metastatic brain tumor. Subsequently, he experienced a third transfer to our hospital, emergency surgery was performed to alleviate cerebral edema and excise a lesion in the left frontal lobe. Postoperative pathology was inconclusive, but a multidisciplinary team meeting, aided by experienced radiologists, eventually confirmed a diagnosis of pulmonary artery sarcoma (PAS) with systemic metastases. This case underscores the necessity of promptly ruling out PAS in patients presenting with significant emboli in the central pulmonary arteries and suggests early referral to specialized centers for suspected cases.

## Introduction

Pulmonary Artery Sarcoma (PAS) is an exceptionally rare vascular malignancy that primarily manifests within the pulmonary arterial lumen, rarely invading the lung parenchyma (1). The clinical presentation of PAS is subtle and nonspecific, featuring symptoms such as dyspnea, cough, chest pain, and syncope, which frequently lead to a delayed diagnosis. As the disease insidiously progresses, pulmonary metastases at diagnosis are more common among younger patients (2), whose robust cardiopulmonary compensatory mechanisms often mask symptoms, thereby prolonging diagnosis time. Furthermore, literature reports indicate that brain metastases, although rare (3), generally emerge post-surgically, underscoring the complex management required for this condition and suggesting a potential for poorer outcomes. This case report details the diagnostic journey of a young male with suspected pulmonary artery intimal sarcoma, characterized by extensive systemic metastases and numerous hospital referrals before confirmation of his condition.

## Case-report

**Figure 1:**
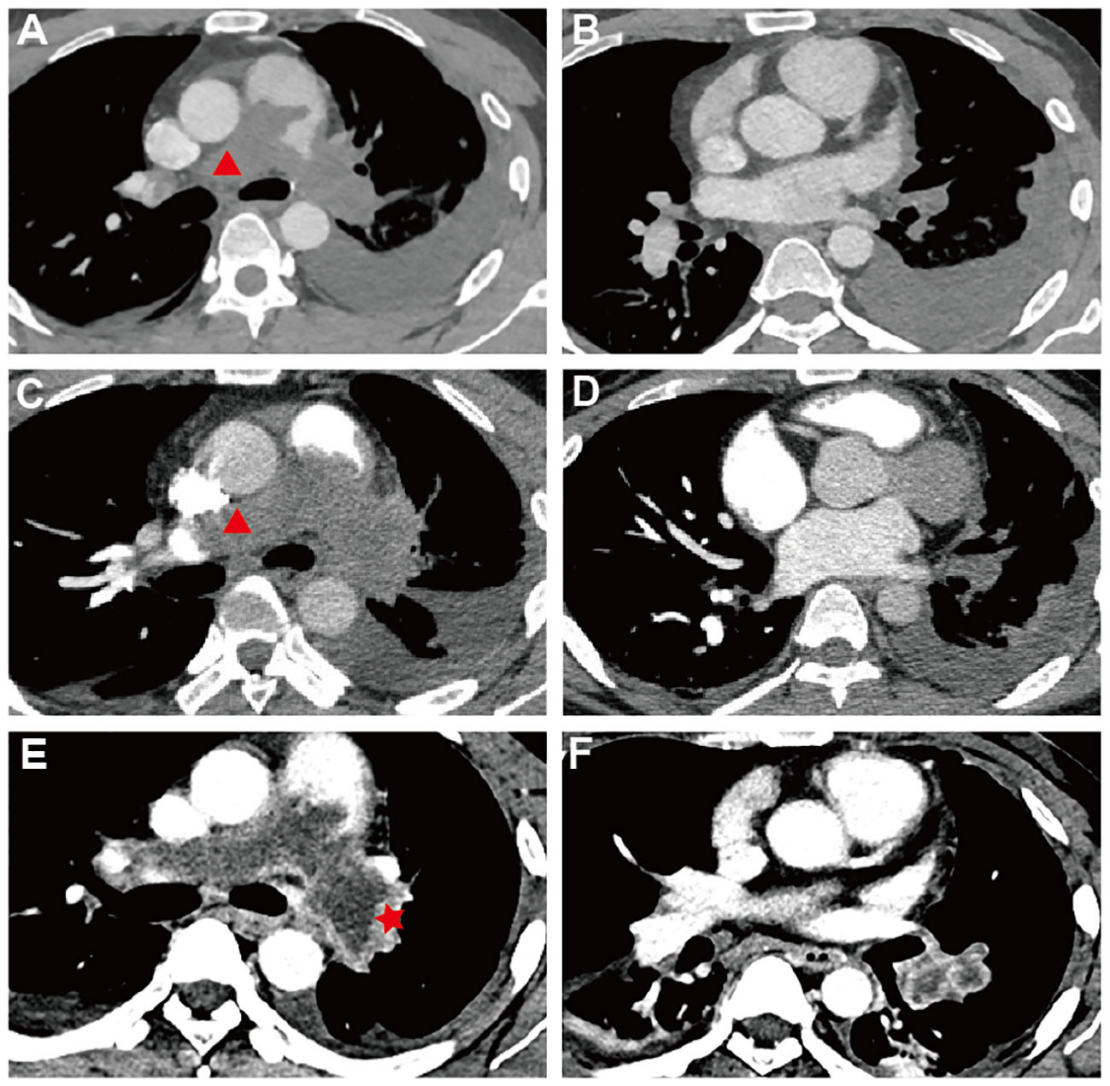
Pulmonary Computed Tomography Angiography| **(A–F)** Sequential PCTA Scans Demonstrate PAS Progression: **(A, B)** October 2022 - Low-density defects fill and expand both pulmonary trunks, eclipsing artery walls (red triangle) and causing left-sided pleural effusion; trunk diameter reaches 35 mm. **(C, D)** December 20–2 - Defects in pulmonary trunks enlarge, extending into the right ventricular outflow tract with progression beyond the left artery. **(E, F)** February 20–3 - Delayed-phase imaging reveals unevenly enhanced, irregular PAS lesions (red start), **(F)** the lesion within the branches of the left lower lobe arteries is markedly enlarged with inhomogeneous enhancement. **(A, C)** The red triangle marks the special PAS characteristics: complete arterial lumen filling with an “eclipsing vessel sign” and vessel expansion.

A 33-year-old male presented at a comprehensive hospital with a persistent, severe dry cough and occasional dyspnea, but no fever or chest pain. Chest radiographs displayed scattered pneumonia and pleural effusion in the left lung. However, anti-infective treatments yielded no significant improvement. Blood gas analysis indicated severe hypoxemia (PaO2 = 62.8 mmHg). Pulmonary computed tomography angiography (PCTA) identified an enlarged pulmonary artery trunk (up to 35 mm) and low-density filling defects in the main and both left and right pulmonary artery trunks (**Figures 1A, B**), which initially suggested a misdiagnosis of pulmonary embolism with pulmonary hypertension—a common interpretation under these circumstances. Furthermore, a Doppler ultrasound of the lower limb found no thrombi, indicating a need for additional explorations for other thrombosis sources or causes. Cytological analysis of the pleural effusion indicated an exudative nature with slightly elevated levels of neuron-specific enolase (NSE), while tests for carcinoembryonic antigen (CEA), squamous cell carcinoma antigen (SCC), and cytokeratin fragment 21–1 (CYFRA21–1) were negative, eliminating concerns of mediastinal lung cancer. Despite initiating anticoagulant therapy, the patient’s symptoms—cough, dyspnea, and newly developed hoarseness—intensified. A follow-up CTPA two months later revealed further enlargement of the pulmonary artery trunk and a progressive increase in pulmonary artery pressure (**Figures 1C, D**).

**Figure 2:**
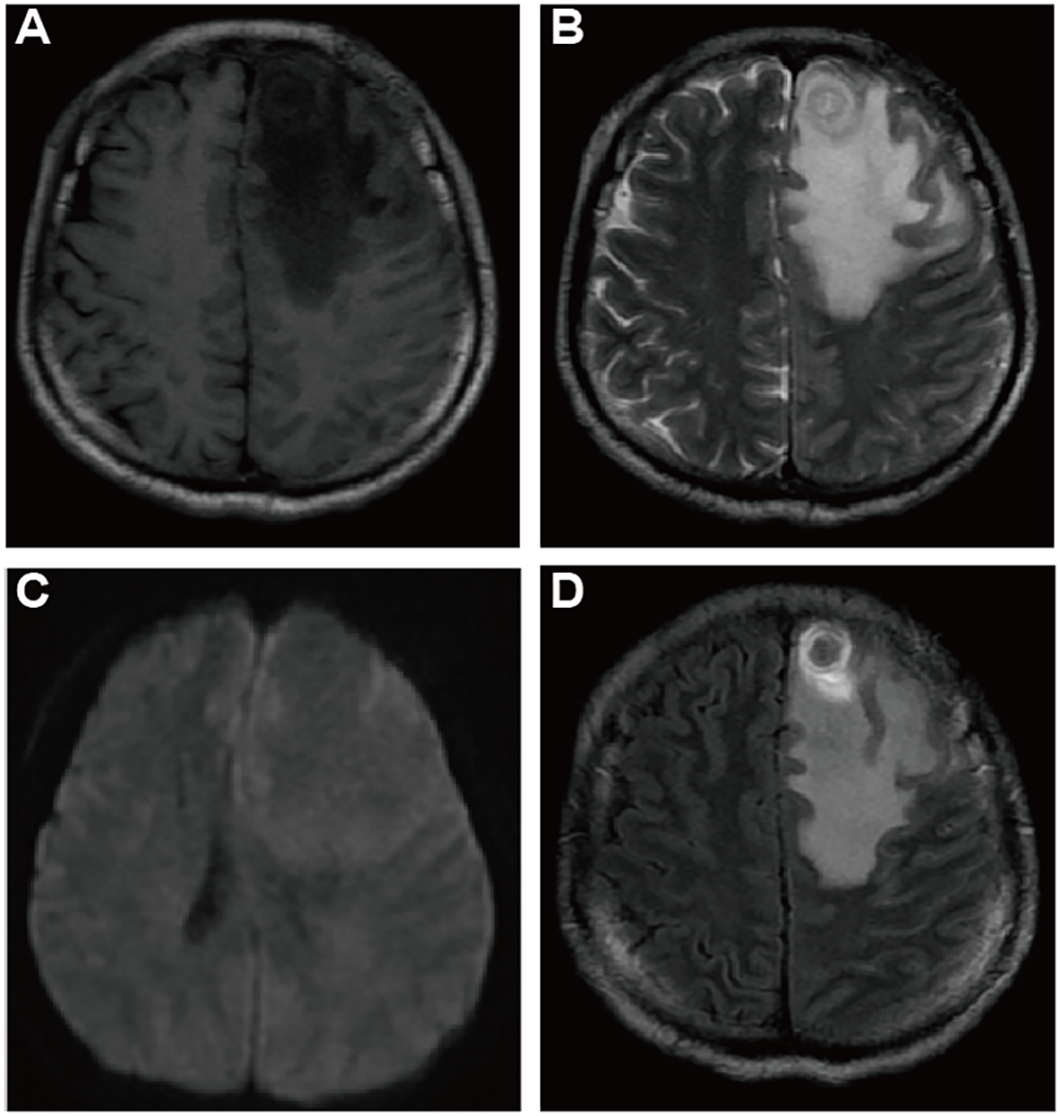
Cranial Magnetic Resonance Imaging (CMRI) of suspected brain metastases: CMRI T1WI **(A)**, T2WI **(B)**, DWI **(C)**, Enhanced T1WI **(D)** axial images demonstrating a suspicious metastatic lesion: a subcortical roundish nodule located in the left frontal lobe, with low signal in T1WI and slightly hyperintense concentric ring pattern and uneven signal in T2WI, with peripheral circumferential ring-like enhancement, surrounded by a large area of edema, no apparent diffusion restriction.

Despite treatment, the patient’s condition deteriorated as his cough intensified and was further complicated by dyspnea, fatigue, hoarseness, and chest tightness. He was subsequently referred to another Cardiovascular Center, where he continued to be treated under the assumption of pulmonary embolism. However, a fortuitous fall that resulted in a head injury led to a pivotal change in his diagnosis. A computed tomography (CT) scan of the head uncovered a large, heterogeneous low-density lesion in the left frontal lobe. Subsequent magnetic resonance imaging (MRI) (**Figures 2A–D**) delineated the lesion as solid tissue with irregular ring enhancement and significant surrounding edema, indicative of an intracranial metastatic tumor. Following these findings, he was urgently transferred to the neurosurgery department of a cancer hospital for emergency surgical intervention.

**Figure 3:**
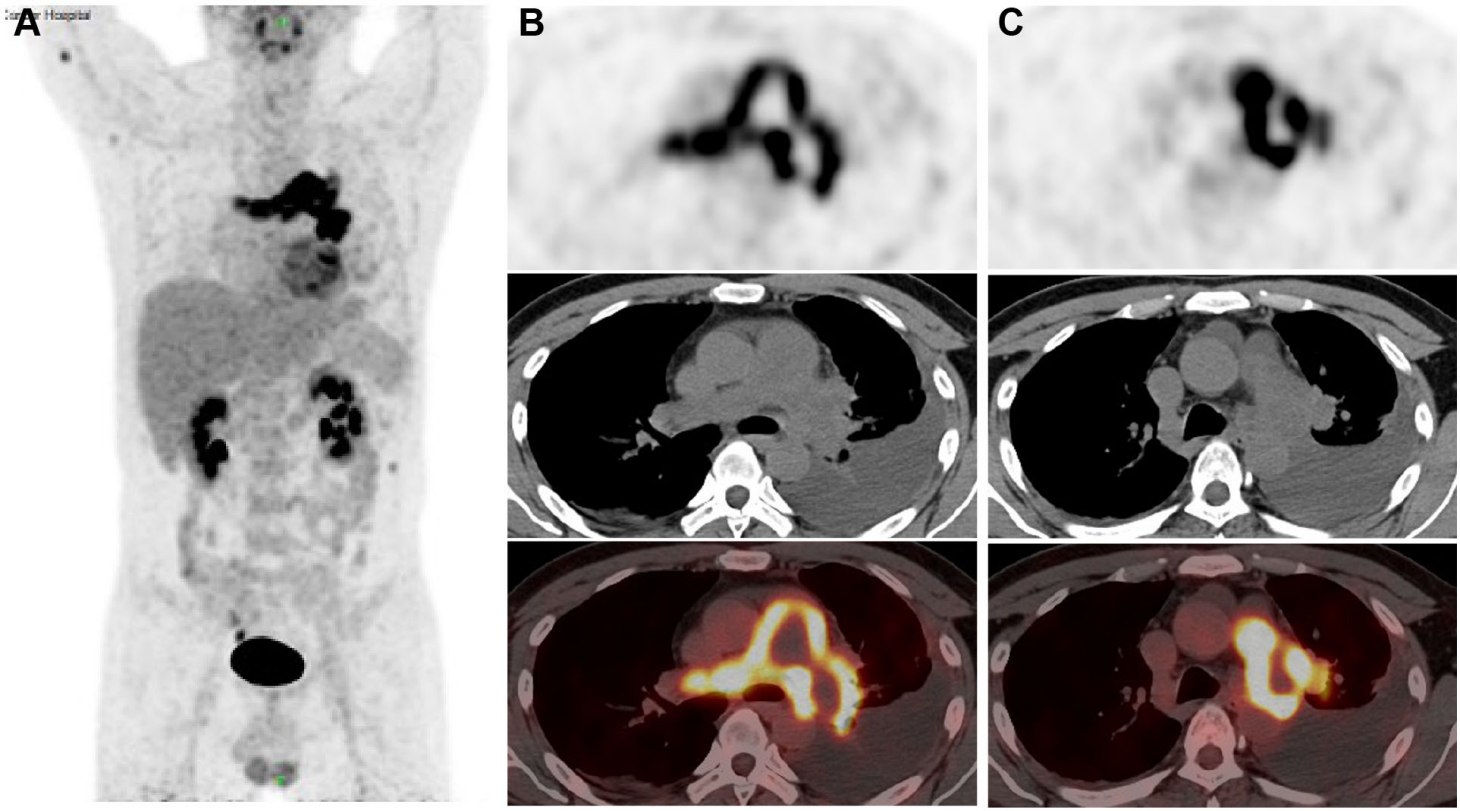
18F-FDG Positron Emission Tomography-Computed Tomography (PET-CT) images **(A)** The anteroposterior 3-dimensional maximum intensity projection image (MIP) demonstrated a hypermetabolic lesion in the left and right artery trunks, while abnormal FDG metabolic nodules were found in the right deltoid muscle and left transversus abdominis muscle; **(B, C)** irregular PAS lesion in the pulmonary artery wall, with increased FDG uptake (SUV max=12.7).

**Figure 4:**
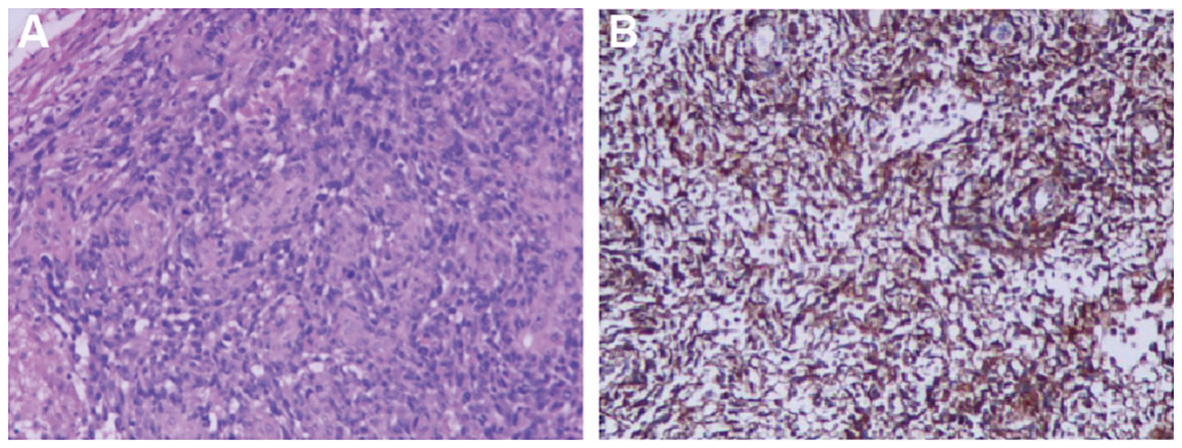
Histopathological Findings **(A)** Irregular tumor cell morphology, spindle-shaped and pleomorphic, with large, deeply stained nuclei showing obvious atypia, frequent nuclear division, and no specific arrangement pattern (×100 for cell morphology). **(B)** Tumor immunohistochemical markers reveal diffusely only positive staining for Vimentin (×100 for immunohistochemistry).

An ^18^F-FDG Positron Emission Tomography - Computed Tomography (PET-CT) scan revealed significant radiotracer uptake in the irregular lesion located in the pulmonary artery trunk (**Figures 3B, C**), with a maximum standardized uptake value (SUV) of 12.7. Additionally, abnormal FDG metabolic nodules were detected in the right lower lobe of the lung, right deltoid, and left transverse abdominis (**Figure 3A**). In the delayed phase of repeated CTPA, the lesions displayed inhomogeneous enhancement— a detail previously missed (**Figures 1E, F**). This finding reinforced the suspicion of a metastatic process extending beyond the initial intracranial diagnosis. Surgical resection of the lesion in the left frontal lobe was undertaken to reduce cerebral edema. Pathological analysis thereafter highlighted epithelioid characteristics in the tumor cells (H&E staining, **Figure 4A**). Furthermore, multiple immunohistochemical tests failed to show differentiation (**Figure 4B**), raising the possibility of an undifferentiated sarcoma or meningioma.

The final diagnosis of undifferentiated pulmonary artery sarcoma with systemic metastases was confirmed at a multidisciplinary team meeting (MDT) at our hospital, involving neurosurgeons, oncologists, radiologists and thoracic surgeons. Initially, examining the intracranial tumor tissue resected during surgery, the pathologist proposed a hypothesis of a poorly differentiated sarcoma or meningioma, based on the indistinct differentiated of the intracranial tumor and its histomorphometric features. However, reflecting on intraoperative observations—the tumor’s location beneath the cerebral cortex and its infiltration into the brain parenchyma—the neurosurgeon dismissed meningioma as a diagnosis. The radiologist highlighted that the tumor in the main trunk of the pulmonary artery was likely a pulmonary artery sarcoma as indicated by the imaging characteristics seen in CTPA and PET-CT scans. Notably, the highest FDG uptake on the PET-CT was located in this main trunk lesion, identifying it as the primary tumor, with additional intracranial and muscular lesions displaying metastatic traits.

The multidisciplinary team consensus was to forego surgery given the patient’s poor prognosis and anticipated challenges with chemotherapy side effects. The team proactively opted for targeted therapy as an alternative treatment strategy. His genetic testing showed CDK4/6 pathway overexpression, leading to the selection of targeted therapy with Palbociclib (125 mg/day, days 1–21 of a 28-day cycle) combined with Temozolomide (20 mg/day, days 1–5 of the same cycle) and localized cranial radiotherapy (60 Gy in 20 fractions). Despite this regimen, the primary tumor continued to enlarge after two treatment cycles, as evidenced by CTPA findings, invading the distal pulmonary arteries and causing localized diffuse dilation. The metastatic lesions also worsened, with new nodules appearing in both lung lobes and pleural regions, alongside noticeable growth in intramuscular metastases. Palbociclib was discontinued due to these findings. One month later, the patient died from respiratory failure caused by progressive disease. However, control over intracranial metastases was effective, with no new metastatic foci appearing, and the patient remained lucid throughout. His overall survival period was 9.1 months after the initial symptoms.

## Discussion

Pulmonary artery sarcoma (PAS) is a rare and extremely malignant tumor that predominantly affects large vessels (4), typically confined to the trunk of the pulmonary artery (1). It is challenging to obtain tissue samples for diagnosis due to its location, hence the diagnosis is usually made after surgery or autopsy (5). Distant metastasis is rare in the reported PAS cases (6). This also means a loss of opportunities for surgical intervention (7). For patients who are ineligible for surgery, multidisciplinary team (MDT) consultations are essential for diagnosis. Correct diagnosis as early as possible can help to provide timely surgical intervention or to try more viable treatment options.

Patients with PAS patients often typically exhibit no symptoms in the initial stages; however, they may later present with signs indicative of pulmonary hypertension, such as dyspnea, chest pain, cough, and syncope. In younger patients, these atypical symptoms often persist unnoticed for a longer period due to more effective cardiorespiratory compensation, leading to widespread involvement of the bilateral pulmonary trunk by the time of diagnosis (7), as illustrated in our case. Multimodal therapy, which includes chemotherapy and radiotherapy following surgical resection, is particularly beneficial for younger patients (7), Therefore, early detection is essential, especially to distinguish these cases from pulmonary embolism (PE), for which Computed Tomography Pulmonary Angiography proves highly effective (8).

Key CTPA indicators for PAS include mass enhancement and a “grape-like appearance”, as observed in this case, alongside complete arterial lumen filling characterized by an “eclipsing vessel sign” and vessel expansion (9–11). Delayed-phase imaging here shows an unevenly enhanced mass, aligning with studies that highlight optimal enhancement during the venous phase of dual-phase contrast-enhanced CT (with pulmonary arterial and venous phase imaging) (12). This imaging technique, also known as delayed scanning, is advocated to best capture the definitive features of mass enhancement. The “grape-like appearance” underscores the intraluminal filling defects that extend to the segmental and sub-segmental arteries, accompanied by local aneurysmal dilatations, which has 100% specificity for diagnosis of PAS, particularly in advanced stages (13). These markers are more evident on MRI due to its superior tissue resolution. Specific MRI sequences, such as fat-suppressed T2-weighted imaging, Diffusion Weighted Imaging (DWI), and dynamic contrast-enhanced sequences, are particularly indicative of PAS, especially in comparison with PE. For patients suspected of having pulmonary embolism but who show poor response to anticoagulation, PET-CT scans provide greater utility than MRI. The ^18^F-FDG PET-CT is recognized as the most effective method to differentiate PAS from pulmonary thromboembolism (14), and it is also capable of identifying metastatic lesions.

Given the rarity of Pulmonary Artery Sarcoma, definitive treatment guidelines have not been established. Surgical resection—typically procedures like pneumonectomy or pulmonary endarterectomy—offers the possibility of extended survival, with a median survival of 20 months (15, 16). However, the overall survival rate post-surgery remains low, and recurrences are common. Postoperative chemotherapy could significantly enhance median survival, potentially increasing life expectancy to up to five years. While the results from solely using chemotherapy or radiotherapy for PAS are generally suboptimal (17), aggressive chemotherapy regimens are advised for those with inoperable or metastatic PAS. A doxorubicin-based regimen is commonly recommended for soft tissue sarcomas (18). Effective combinations include doxorubicin with ifosfamide (19), and ifosfamide with epirubicin (20). Moreover, Paclitaxel has been shown to be effective (18, 21), especially in patients prone to developing pulmonary artery hypertension. Recent advancements in anticancer therapies have led to the exploration of vascular-targeting agents like apatinib, anlotinib, and PD-1/PD-L1 immunotherapies in treating PAS, with promising results reported in several studies (22). Nonetheless, a standardized chemotherapy or targeted treatment protocol for PAS has not yet been established.

In conclusion, radiotherapy, chemotherapy and targeted therapy could be attempted in patients with pulmonary artery sarcoma that is unresectable or recurrent, although the most effective way of prolonging survival has been surgical intervention. Early diagnosis, which allows for multimodal treatment significantly extends patient survival chances, is critically dependent on physicians’ comprehensive understanding of PAS. We reported this rare case where PAS was first identified via a brain metastatic lesion, emphasizing the crucial need for vigilance in recognizing diagnostic signs of PAS even in its metastatic stages.

## Data availability statement

The raw data supporting the conclusions of this article will be made available by the authors, without undue reservation.

## Ethics statement

The studies involving humans were approved by Ethics Committee of Kunming Yan’an Hospital. The studies were conducted in accordance with the local legislation and institutional requirements. The participants provided their written informed consent to participate in this study. Written informed consent was obtained from the individual(s) for the publication of any potentially identifiable images or data included in this article.

## Author contributions

NT: Writing – original draft, Writing – review & editing. ZO: Writing – review & editing. XD: Investigation, Writing – review & editing. XZ: Methodology, Writing – review & editing. YZ: Writing – review & editing. JC: Data curation, Writing – review & editing. DL: Writing – review & editing. HD: Supervision, Writing – review & editing. CL: Funding acquisition, Supervision, Writing – review & editing.
